# Supplementary material for: Detection of Hydroxychloroquine Retinopathy via Hyperspectral and Deep Learning through Ophthalmoscope Images
Source: Diagnostics (Basel). 2023 Jul 14;13(14):2373. doi: 10.3390/diagnostics13142373 (PMC10377856; doi:10.3390/diagnostics13142373)
Supplement: Supplementary file 1 [file diagnostics-13-02373-s001.zip › diagnostics-2432435-supplementary.pdf]

diagnostics

*Supplementary Materials*

# Detection of Hydroxychloroquine Retinopathy via Hyperspectral and Deep Learning Through Ophthalmoscope Images

**Wen-Shuang Fan [1], Hong-Thai Nguyen [2], Ching-Yu Wang [1], Shih-Wun Liang [2], Yu-Ming Tsao [2], Fen-Chi Lin [3,*] and Hsiang-Chen Wang [2,4,*]**

[1] Department of Ophthalmology, Dalin Tzu Chi Hospital, Buddhist Tzu Chi Medical Foundation, Chiayi 62247, Taiwan; wsfan@tzuchi.com.tw (W.-S.F.), s19001052@gmail.com (C.-Y.W.)
[2] Department of Mechanical Engineering, National Chung Cheng University, Chiayi 62102, Taiwan; nguyenhongthai194@gmail.com
[3] Department of Ophthalmology, Kaohsiung Armed Forces General Hospital, Kaohsiung 80284, Taiwan; eses.taiwan@gmail.com
[4] Director of Technology Development, Hitspectra Intelligent Technology Co., Ltd, Kaohsiung 80661, Taiwan
* Correspondence: eses.taiwan@gmail.com (F.-C.L.); hcwang@ccu.edu.tw (H.-C.W.)

## S1. Ophthalmoscope

An ophthalmoscope is an ophthalmic diagnostic optical instrument used to observe and record the condition of the fundus. It can record and preserve fundus images in black and white or color photos, and its optical design is based on the optical principle of Gullstrand's nonreflective indirect ophthalmoscope, which can directly capture the retina. Ophthalmologists can diagnose retinal diseases by using fundus images.

The human fundus does not glow even despite the fact that various types of light enter the eyeball. Light can illuminate the fundus but is weak and is, thus, inadequate for observing the fundus, rendering photography impossible. The reflected light of the cornea of a human eye is much brighter than that of the fundus and interferes with the observation of the fundus. Therefore, a fundus camera must include an illumination system that brightens the fundus with strong light and an imaging system that prevents the effects of intense reflections on the cornea. A complete fundus camera optical system consists of a camera, lighting, and observation systems. Figure S1 is a diagram of a Zeiss fundus camera [40]. The Xenon flash light source is collected by the *L1* concentrator and passes through a series of lenses and mirrors to the eyes of a patient. According to the principle of Gullstrand, *M2* is a circular mirror, and thus, the input of the light source is separated from the path of observation. In the case that an input source is reflected by the retina, the source passes through the center of the mirror and displays a fundus image on the camera's back sheet. Mirrors *M3, M4,* and *L7* and the independent light source *S1* form an observation system that can observe and monitor a fundus image in real time.

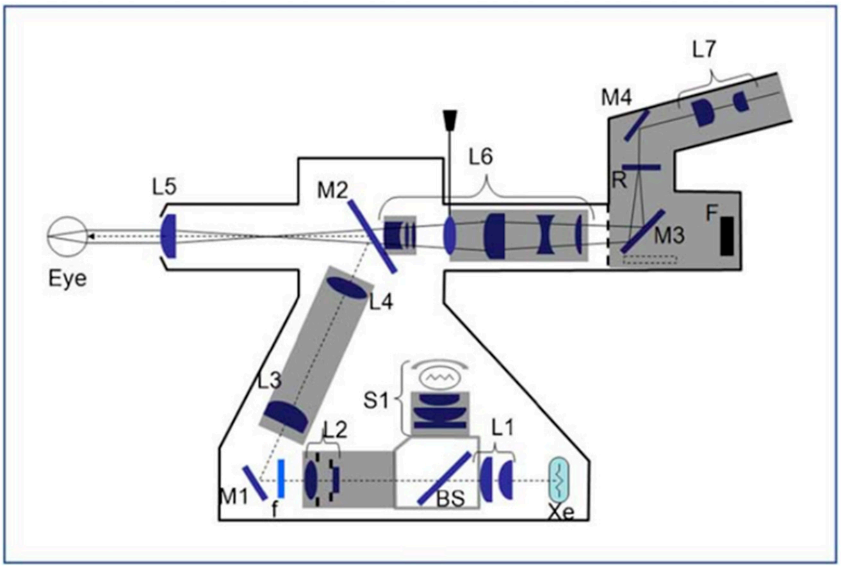


**Figure S1.** An image of the Zeiss fundus camera. *Xe* is xenon flash, *L1* is concentrator, *S1* is observation system light source, *L2-7* is photographic system optics, *BS* is beam splitter, *f* is filter, *M1-4* is mirror, *F* is camera negative, *R* is targeting, and the dotted and solid lines are the light source input and observation paths, respectively [40].

## S2. Hyperspectral Conversion Algorithm

In this study, the hyperspectral conversion algorithm aims to determine the relationship between the ophthalmoscope (Kowa Nonmyd 7) and the spectrometer (Ocean Optics, QE65000) by shooting a common target. We use the standard 24-color card (X-Rite Classic, 24 Color Checkers) as a common target, which contains common colors in nature, and most of them represent natural objects, such as human skin, leaves, and blue sky. We then use the fundus mirror and spectrometer to measure the spectrum of the 24-color card and take images. The obtained images are converted into a set of sRGB channel values, and the transformation matrix is obtained using the two sets of data. The complete process of modeling is shown in Figure S2.

When modeling, the spectral and image data collected above must be converted to the same color gamut space (CIE 1931 XYZ color space). The detailed processing processes of the ophthalmoscope and spectrometer will be described below. The color gamut space of the image (JPEG, 24 Bit Depth) obtained by the ophthalmoscope end during shooting is sRGB (0 ~ 255), and the conversion of sRGB into XYZ color gamut space is shown in Equation (S1). From this equation, we can convert sRGB into XYZ color gamut space and $XYZ_{Funducopy}$ under the measurement light source.

$$\begin{bmatrix} X \\ Y \\ Z \end{bmatrix} = [M_A][T]\begin{bmatrix} f(R_{sRGB}) \\ f(G_{sRGB}) \\ f(B_{sRGB}) \end{bmatrix} \times 100\,, 0 \leqq \begin{matrix} R_{sRGB} \\ G_{sRGB} \\ B_{sRGB} \end{matrix} \leqq 1, \tag{S1}$$

Before converting to the XYZ color gamut space, it must be normalized into sRGB (0–1) and converted into linear RGB through the Gamma function, as shown in Equation (S2).

$$f(n) = \begin{cases} \left(\frac{n+0.055}{1.055}\right)^{2.4}, & n > 0.04045 \\ \left(\frac{n}{12.92}\right), & otherwise \end{cases}, \tag{S2}$$

The linear RGB is converted into the XYZ color gamut space through the transformation matrix, as shown in Equation (S3).

$$[T] = \begin{bmatrix} 0.4104 & 0.3576 & 01805 \\ 0.2126 & 0.7152 & 0.0722 \\ 0.0193 & 0.1192 & 0.9505 \end{bmatrix}, \tag{S3}$$

However, during the conversion, because the sRGB color gamut space defines the White point as the D65 light source ($X_{CW}$, $Y_{CW}$, $Z_{CW}$) rather than the white point of the measurement light source ($X_{SW}$, $Y_{SW}$, $Z_{SW}$), so we adopt the conversion matrix through the color. $M_A$ is used to convert the white point of the D65 light source into the white point of the measurement light source, as shown in Equation (S4).

$$[M_A] = \begin{bmatrix} X_{SW}/X_{CW} & 0 & 0 \\ 0 & Y_{SW}/Y_{CW} & 0 \\ 0 & 0 & Z_{SW}/Z_{CW} \end{bmatrix}, \tag{S4}$$

On the spectrometer side, converting the obtained spectral data R(λ) (380–780 nm, 1 nm) into the XYZ color gamut space requires the light source spectrum S(λ) when using the ophthalmoscope to shoot and the XYZ color matching function $\bar{x}(\lambda)$, $\bar{y}(\lambda)$, $\bar{z}(\lambda)$ (Color matching functions, CMF), as shown in Figure S3. The Y value of the XYZ color gamut space is proportional to the brightness. The Y value of the light source spectrum (maximum brightness) and its upper limit are set to 100 to obtain the brightness ratio k, as shown in Equation (S5).

$$k = 100/\int_{380nm}^{780nm} S(\lambda)\bar{y}(\lambda)\ d\lambda, \tag{S5}$$

Finally, through Equations (S6)–(S8), the spectral data are converted into the XYZ value ($XYZ_{Spectrum}$) regulated in the XYZ color gamut space.

$$X = k\int_{380nm}^{780nm} S(\lambda)R(\lambda)\bar{x}(\lambda)\ d\lambda, \tag{S6}$$

$$Y = k\int_{380nm}^{780nm} S(\lambda)R(\lambda)\bar{y}(\lambda)\ d\lambda, \tag{S7}$$

$$Z = k\int_{380nm}^{780nm} S(\lambda)R(\lambda)\bar{z}(\lambda)\ d\lambda, \tag{S8}$$

After obtaining $XYZ_{Funducopy}$ and $XYZ_{Spectrum}$, error factors, such as nonlinear response, dark current, and color shift, may occur when the ophthalmoscope is shooting. Therefore, we finally obtain the correction matrix C that can be used to correct the ophthalmoscope through multiple regression analysis, as shown in Equation (S9).

$$[C] = \left[XYZ_{Spectrum}\right] \times pinv([V]), \tag{S9}$$

The spectrometer has a linear response, so we use the Y value (brightness) of the 19th to 24th color blocks in the 24-color cards taken by the ophthalmoscope and the spectrometer to perform linear regression analysis by calculating the Y value of $XYZ_{Funduscopy}$ and $XYZ_{Spectrum}$ (Linear regression). As shown in Figure S4, the ophthalmoscope has a nonlinear response, and its coefficient of determination R2 (Coefficient of determination) reaches 0.9725 in the third-order linear regression, which is quite similar, so we speculate that the ophthalmoscope is nonlinear. The response can be corrected by a third-order equation, and the nonlinear response correction variable is defined as $V_{Non\text{-}linear}$, as shown in Equation (S10).

$$V_{Non-linear} = [X^3\ Y^3\ Z^3\ X^2\ Y^2\ Z^2\ X\ Y\ Z\ 1]^T, \tag{S10}$$

In the part of the dark current, the dark current is expressed as a constant because it does not change with the amount of light, so the dark current correction variable is defined as $V_{Dark}$, as shown in Equation (S11).

$$V_{Dark} = [\alpha], \tag{S11}$$

In the color shift part, a problem with color matching occurs. The relationship between X, Y, and Z values needs to be considered because the ophthalmoscope image has been converted into the XYZ color gamut space. As shown in the XYZ color matching function in Figure S3, $\bar{x}$, $\bar{y}$, and $\bar{z}$ are related in spectral distribution, so all possibilities among X, Y, and Z are listed and defined as $V_{Color}$, as shown in Equation (S12).

$$V_{Color} = [XYZ\ XY\ YZ\ XZ\ X\ Y\ Z]^T, \tag{S12}$$

After considering all the errors, we use $V_{Color}$ as the base to multiply $V_{Non\text{-}linear}$ for nonlinear response correction. The result is standardized in the third order to avoid over-correction, and $V_{Dark}$ is added to obtain the variable matrix V, as shown in Equation (S13). Finally, we bring the obtained variable matrix V back to Equation 9 to obtain the correction matrix C.

$$V = [X^3\ Y^3\ Z^3\ X^2Y\ X^2Z\ Y^2Z\ XY^2\ XZ^2\ YZ^2 \\ XYZ\ X^2\ Y^2\ Z^2\ XY\ XZ\ YZ\ X\ Y\ Z\ \alpha]^T, \tag{S13}$$

Through Equation (S9), we extend $XYZ_{Funduscopy}$ to V matrix to obtain corrected X, Y, and Z values ($XYZ_{Correct}$), as shown in Equation (S14).

$$[XYZ_{Correct}] = [C] \times [V], \tag{S14}$$

Given that the wavelength band used in this study is part of visible light (380 nm ~ 780 nm), the correction result can be expressed via chromatic aberration. This study uses CIEDE 2000 to calculate chromatic aberration, so the method uses hue rotation, neutral color compensation, brightness compensation, chromaticity compensation, and tone compensation to solve the problem of inconsistent sensitivity of human eyes to different colors.

CIEDE 2000 needs to convert $XYZ_{Correct}$ and $XYZ_{Spectrum}$ to Lab color gamut space before calculation, as shown in Equations (S15)–(S18).

$$L^* = 116 f\left(\frac{Y}{Y_n}\right) - 16, \tag{S15}$$

$$a^* = 500\left[f\left(\frac{X}{X_n}\right) - f\left(\frac{Y}{Y_n}\right)\right], \tag{S16}$$

$$b^* = 500\left[f\left(\frac{X}{X_n}\right) - f\left(\frac{Y}{Y_n}\right)\right], \tag{S17}$$

$$f(n) = \begin{cases} n^{\frac{1}{3}} & , n > 0.008856 \\ 7.787n + 0.137931 & , otherwise \end{cases}, \tag{S18}$$

According to the results of CIEDE 2000 chromatic aberration calculation, the chromatic aberration between the ophthalmoscope and the spectrometer is very obvious before correction. The average color difference reaches 21.4; after correction, the average color difference between the two decreases to 4.07, and the difference can be seen visually, as shown in Figure S5.

To convert the XYZ value ($XYZ_{Correct}$) obtained after ophthalmoscope correction into spectral information, we conduct principal component analysis (PCA) on the reflection spectrum information ($R_{Spectrum}$) of the standard 24-color card to obtain the reflection spectrum of the 24-color card the principal components and their corresponding principal component scores (Score, eigenvalues). We subject the principal component scores and $XYZ_{Correct}$ to multiple regression analysis and finally obtain the transformation matrix M.

We perform principal component analysis on $R_{Spectrum}$ and found that the first 12 sets of principal components (EV) have fully expressed 99.99% of the data variation, as shown in Figure S6. Thus, we use these 12 sets of principal components for dimensionality reduction to obtain principal component scores, carry out multivariate regression analysis based on this data, and use $V_{Color}$ as a variable because $V_{Color}$ has listed all the correlations of X, Y, and Z in detail. We obtain the transformation matrix M between the ophthalmoscope and the spectrometer, as shown in Equation (S19), and perform spectral conversion on $XYZ_{Correct}$ to obtain the analog spectral value $S_{Spectrum}$, as shown in Equation (S20).

$$[M] = [Score] \times pinv([V_{Color}]), \tag{S19}$$

$$[S_{Spectrum}]_{380-780nm} = [EV][M][V_{Color}], \tag{S20}$$

After obtaining the simulated spectrum, we compare it with the reflection spectrum ($R_{Spectrum}$) of the 24-color card. By calculating the Root-mean-square error (RMSE) between the two, the average RMSE is 0.080, and the color blocks 13 to 18 represent the difference between the simulated spectrum and the 24-color card spectrum, as shown in Figure S7, and the simulated spectrum is converted into the Lab color gamut space for CIEDE 2000 color difference comparison. The average color difference is 4.07, as shown in Figure S8.

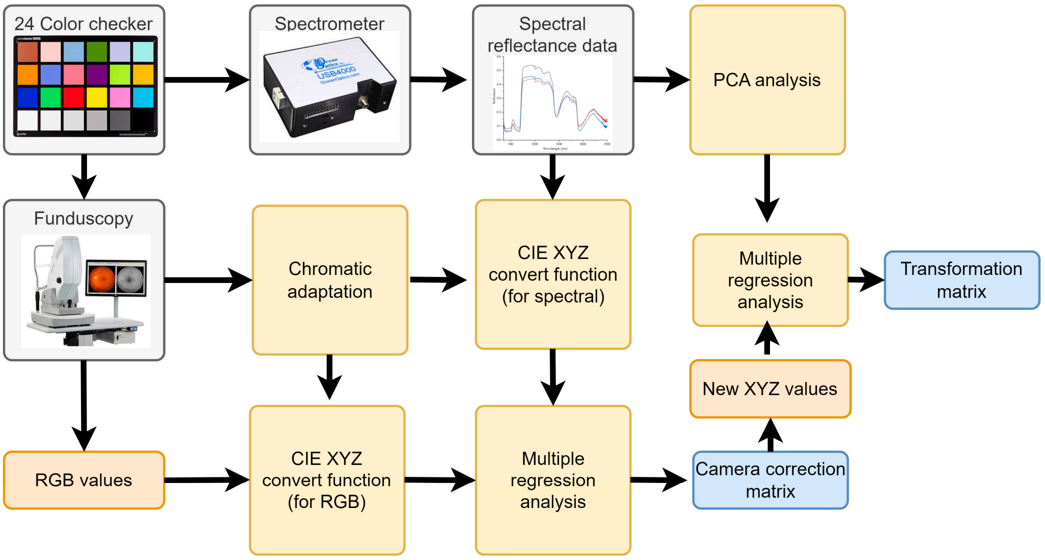


**Figure S2.** Hyperspectral conversion construction flow chart using the standard 24 color blocks (X-Rite Classic, 24 Color Checkers) as the common target for the conversion of the ophthalmoscope and the spectrometer, and converts the ophthalmoscope image into 401 bands of visible spectrum information.

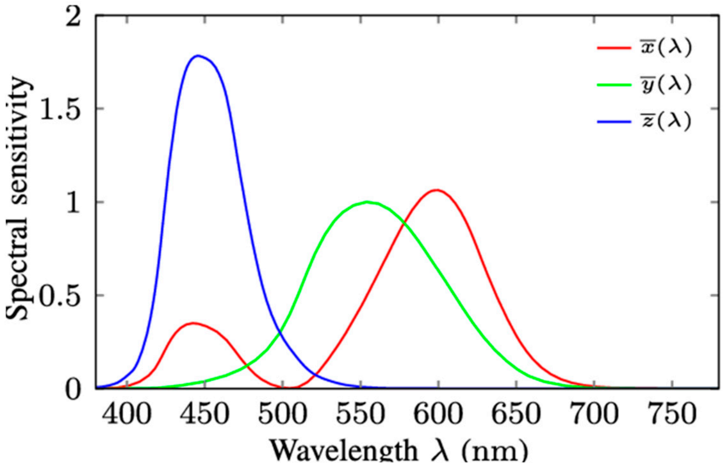


**Figure S3.** XYZ color matching function.

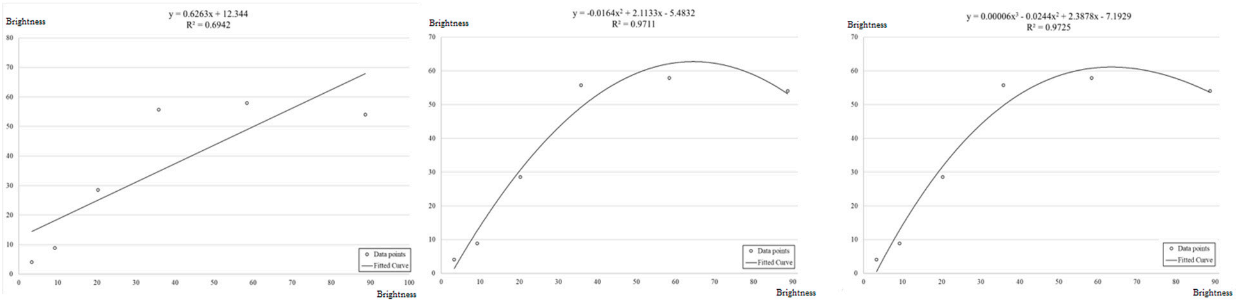


**Figure S4.** Polynomial regression graph of $XYZ_{Funducopy}$ and $XYZ_{Spectrum}$.

| Ophthalmoscope measurement | | | | | | |
|---|---|---|---|---|---|---|
| Color block No. | before correction | spectrometer | chromatic aberration | after correction | spectrometer | chromatic aberration |
| 1 | | | 31.37 | | | 7.71 |
| 2 | | | 15.55 | | | 5.37 |
| 3 | | | 31.77 | | | 2.85 |
| 4 | | | 28.81 | | | 1.65 |
| 5 | | | 25.70 | | | 2.79 |
| 6 | | | 21.13 | | | 2.41 |
| 7 | | | 11.55 | | | 4.74 |
| 8 | | | 32.17 | | | 5.52 |
| 9 | | | 19.28 | | | 1.44 |
| 10 | | | 20.24 | | | 8.16 |
| 11 | | | 14.78 | | | 1.41 |
| 12 | | | 14.20 | | | 2.85 |
| 13 | | | 15.34 | | | 4.08 |
| 14 | | | 27.55 | | | 1.21 |
| 15 | | | 20.44 | | | 2.89 |
| 16 | | | 9.09 | | | 3.57 |
| 17 | | | 20.23 | | | 1.19 |
| 18 | | | 36.45 | | | 1.60 |
| 19 | | | 12.33 | | | 9.41 |
| 20 | | | 14.47 | | | 3.39 |
| 21 | | | 22.67 | | | 7.12 |
| 22 | | | 27.03 | | | 2.50 |
| 23 | | | 25.05 | | | 7.89 |
| 24 | | | 16.33 | | | 5.98 |
| | | Average | 21.40 | | Average | 4.07 |

**Figure S5.** Comparison of chromatic aberration between ophthalmoscope before and after correction and spectrometer.

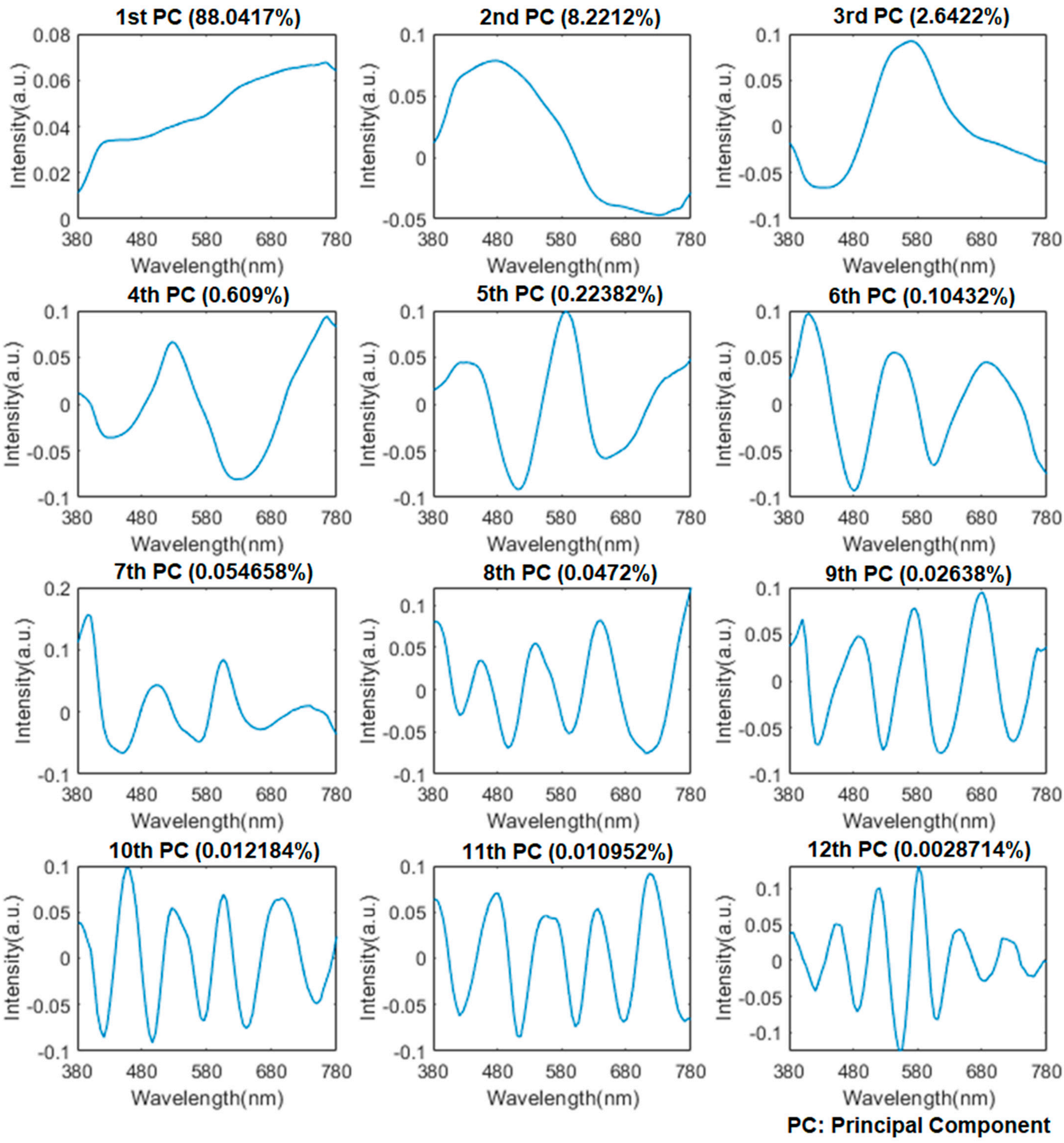


**Figure S6.** Twelve principal components of $R_{Spectrum}$.

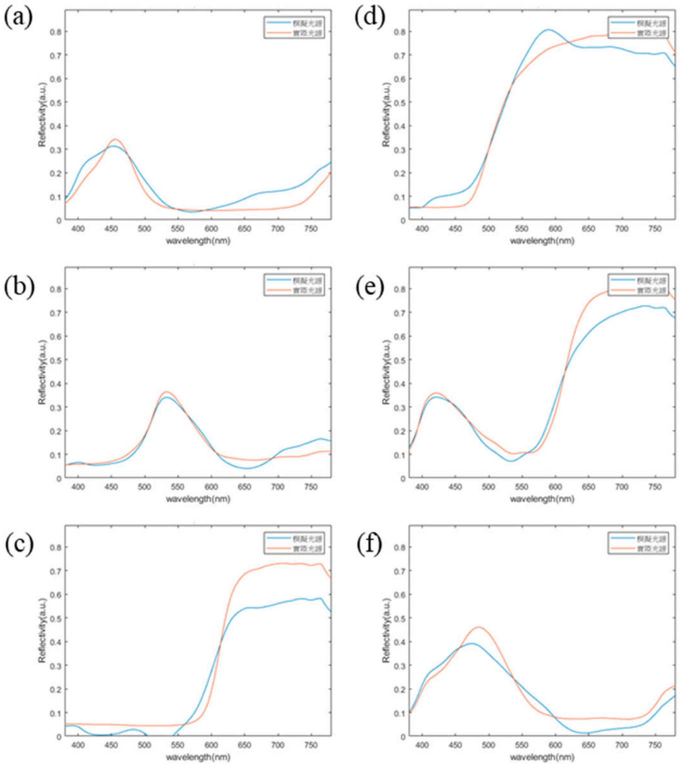


| Color No. | corresponding graph | RMSE |
|---|---|---|
| 1 | - | 0.051 |
| 2 | - | 0.067 |
| 3 | - | 0.112 |
| 4 | - | 0.096 |
| 5 | - | 0.091 |
| 6 | - | 0.042 |
| 7 | - | 0.060 |
| 8 | - | 0.039 |
| 9 | - | 0.065 |
| 10 | - | 0.144 |
| 11 | - | 0.014 |
| 12 | - | 0.048 |
| 13 | (a) | 0.045 |
| 14 | (b) | 0.026 |
| 15 | (c) | 0.087 |
| 16 | (d) | 0.058 |
| 17 | (e) | 0.062 |
| 18 | (f) | 0.051 |
| 19 | - | 0.335 |
| 20 | - | 0.034 |
| 21 | - | 0.207 |
| 22 | - | 0.124 |
| 23 | - | 0.033 |
| 24 | - | 0.026 |
| Average error | | 0.080 |

**Figure S7.** Root Mean Square Error of $S_{Spectrum}$ and $R_{Spectrum}$.

| Color blocks | measured spectrum | | | color display | simulated spectrum | | | color display | color difference |
|---|---|---|---|---|---|---|---|---|---|
| | L* | a* | b* | | L* | a* | b* | | |
| 1 | 45.016 | 36.80 | 33.01 | | 38.683 | 24.80 | 25.98 | | 7.71 |
| 2 | 70.915 | 38.36 | 29.88 | | 67.693 | 32.12 | 33.71 | | 5.39 |
| 3 | 52.567 | 5.83 | -9.17 | | 49.764 | 5.64 | -8.36 | | 2.85 |
| 4 | 42.819 | -6.15 | 28.46 | | 42.363 | -4.54 | 30.12 | | 1.65 |
| 5 | 54.380 | 18.57 | -9.82 | | 56.213 | 21.88 | -8.79 | | 2.78 |
| 6 | 70.376 | -14.16 | 13.97 | | 69.557 | -17.74 | 14.96 | | 2.39 |
| 7 | 59.025 | 47.05 | 69.79 | | 64.437 | 45.86 | 70.93 | | 4.73 |
| 8 | 34.505 | 25.42 | -33.42 | | 39.658 | 18.18 | -28.63 | | 5.52 |
| 9 | 54.822 | 55.34 | 31.88 | | 54.577 | 59.58 | 32.07 | | 1.44 |
| 10 | 26.276 | 25.15 | -20.66 | | 31.387 | 30.57 | -10.24 | | 8.14 |
| 11 | 69.960 | -9.07 | 67.76 | | 71.731 | -8.41 | 67.26 | | 1.41 |
| 12 | 75.208 | 30.22 | 84.32 | | 74.084 | 33.13 | 78.99 | | 2.86 |
| 13 | 32.210 | 22.74 | -33.58 | | 28.540 | 22.79 | -38.79 | | 4.10 |
| 14 | 53.899 | -28.56 | 44.37 | | 54.172 | -28.96 | 41.55 | | 1.21 |
| 15 | 45.456 | 65.66 | 46.94 | | 45.354 | 66.50 | 40.98 | | 2.91 |
| 16 | 86.112 | 20.98 | 78.88 | | 83.210 | 20.63 | 91.07 | | 3.56 |
| 17 | 54.968 | 60.36 | 4.90 | | 53.954 | 62.06 | 3.86 | | 1.18 |
| 18 | 48.794 | -20.35 | -14.16 | | 48.999 | -18.08 | -14.89 | | 1.62 |
| 19 | 81.321 | 17.31 | 25.23 | | 95.471 | 18.42 | 21.42 | | 9.40 |
| 20 | 85.753 | 16.41 | 16.50 | | 80.967 | 16.28 | 18.11 | | 3.39 |
| 21 | 74.915 | 11.41 | 17.08 | | 66.381 | 13.69 | 15.19 | | 7.11 |
| 22 | 54.365 | 10.50 | 13.63 | | 52.181 | 11.35 | 12.53 | | 2.50 |
| 23 | 35.330 | 2.51 | 12.08 | | 36.449 | 8.42 | 9.22 | | 7.90 |
| 24 | 21.295 | 12.34 | 5.77 | | 21.375 | 6.10 | 5.99 | | 5.96 |
| | | | | | | | | average | 4.07 |

**Figure S8.** Chromatic difference diagram of measured spectrum and simulated spectrum of 24 color blocks.

## S3. Comparison of normal retinal spectra by age interval

In this study, we utilize normal fundus mirror images without any lesions for comparative analysis of spectra based on age classification (Figure S9). Additionally, we considered the potential effect of diabetic retinopathy on the spectral analysis of dementia patients and therefore conducted a comparative analysis of different stages of diabetic retina. The corresponding findings are presented in Figure S10.

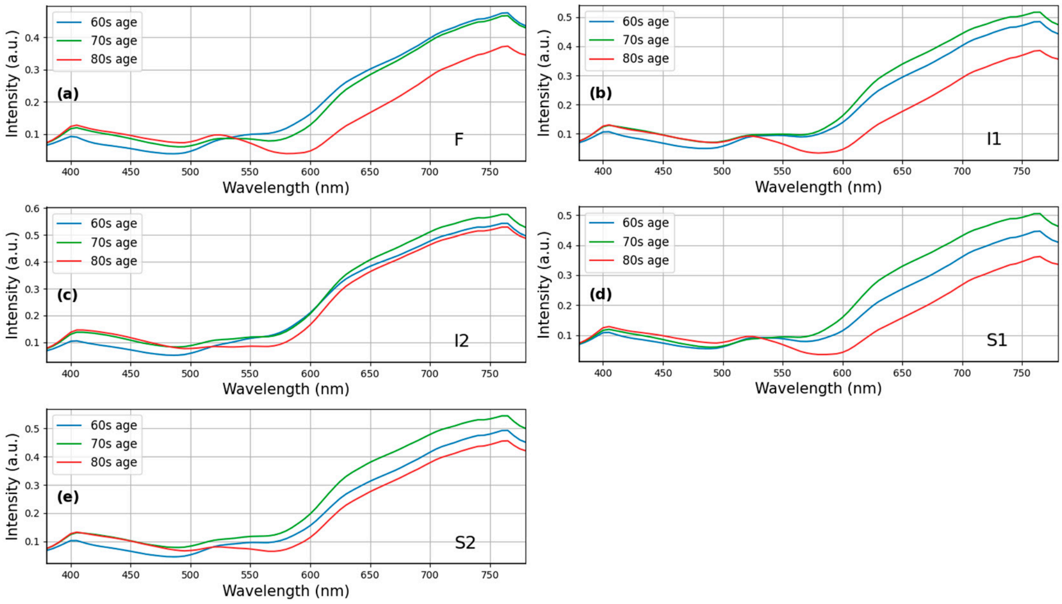


**Figure S9.** Comparison of normal retinal spectra by age interval (**a**) F, (**b**) I1, (**c**) I2, (**d**) S1, and (**e**) S2.

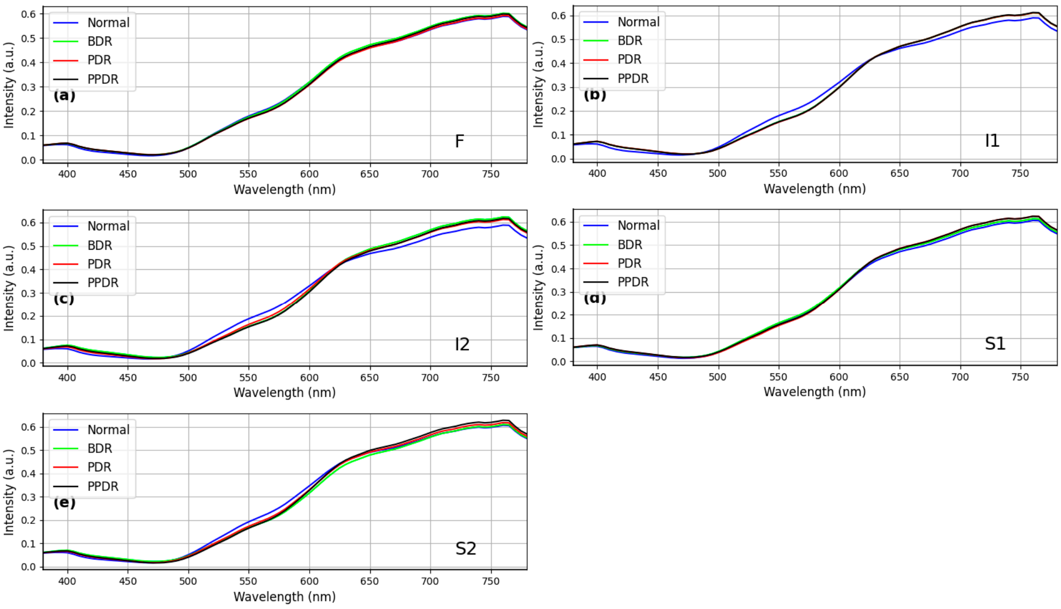


**Figure S10.** Comparison of Diabetic Retina Spectrum by stages (**a**) F, (**b**) I1, (**c**) I2, (**d**) S1, and (**e**) S2.

## S4. Neural Network Models

### S4.1. Dataset partition

Before starting neural network model training, the dataset must be divided into training and test sets. The dataset comprised 66 normal fundus color images and 110 fundus color images of patients who were administered with HCQ. Normal fundus images were obtained from people without HCQ treatment, without dementia, without diabetic retinopathy, and with normal vision. The training set uses Normal 53, HCQ 88, and the test set uses normal (Normal) 13 and HCQ 22.

### S4.2. Neural network model construction and training

This study uses PyTorch as its deep learning framework and uses neural network models including ResNet50, Inception_v3, GoogLeNet, and EfficientNet. Considering that the data obtained are relatively small in deep learning, this study uses transfer learning (Transfer learning). To improve the accuracy, we used the diabetic retinopathy fundus images provided by the Asia Pacific Tele-Ophthalmology Society (APTOS) on a data analysis competition platform (Kaggle) in 2019 for pre-training and then divided the data sets into the four models. We use the loss function as the cross entropy loss function (CrossEntropyLoss). After each Epoch, the loss value can be gradually reduced to adjust the weight. We set the batch size (batch size) to 16, the number of times (Epoch) to 50, and the initial learning rate (Learning rate) to 0.001. Every seven times of training is reduced to 0.1 times the original.

### S4.3. Training performance

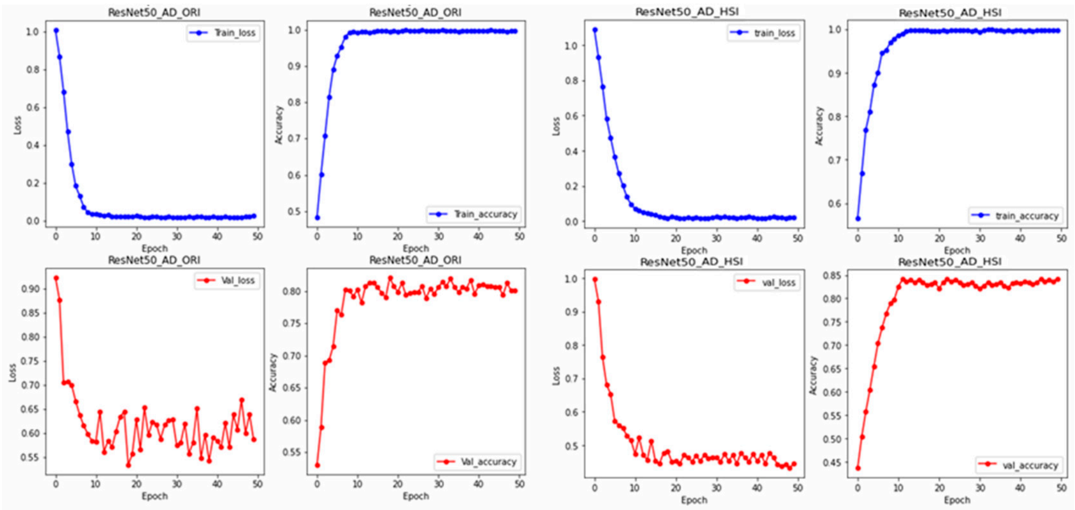


**Figure S11**. Loss and accuracy of ResNet50.

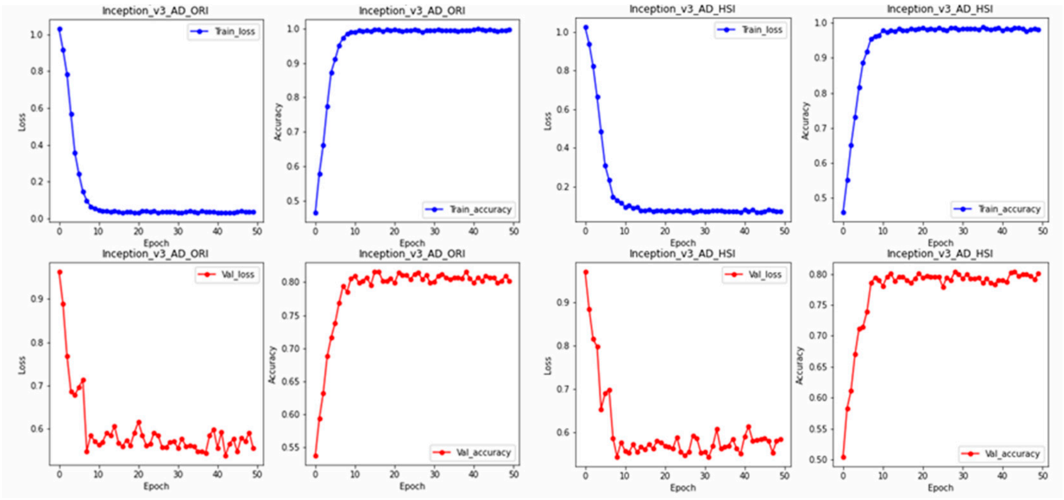


**Figure S12**. Loss and accuracy of Inception_v3

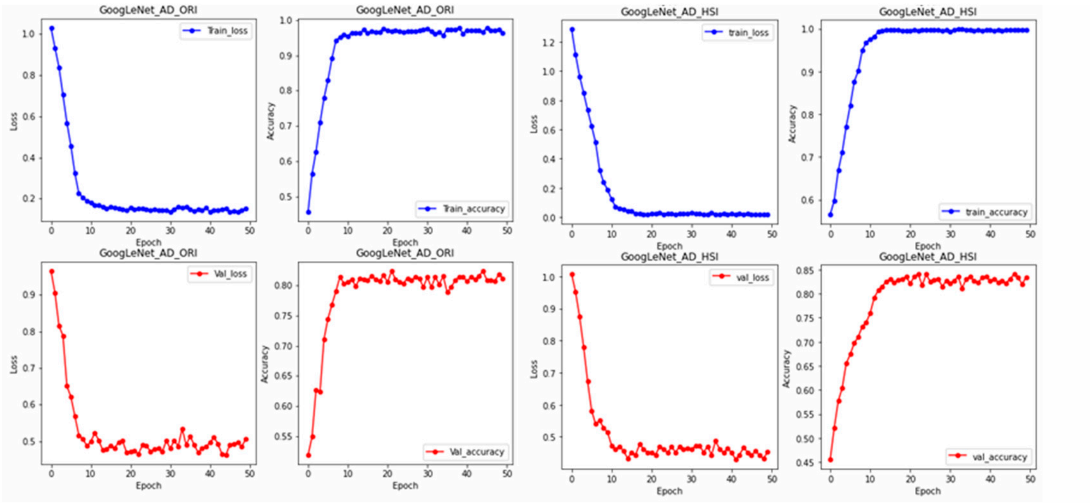


**Figure S13**. Loss and accuracy of GoogLeNet

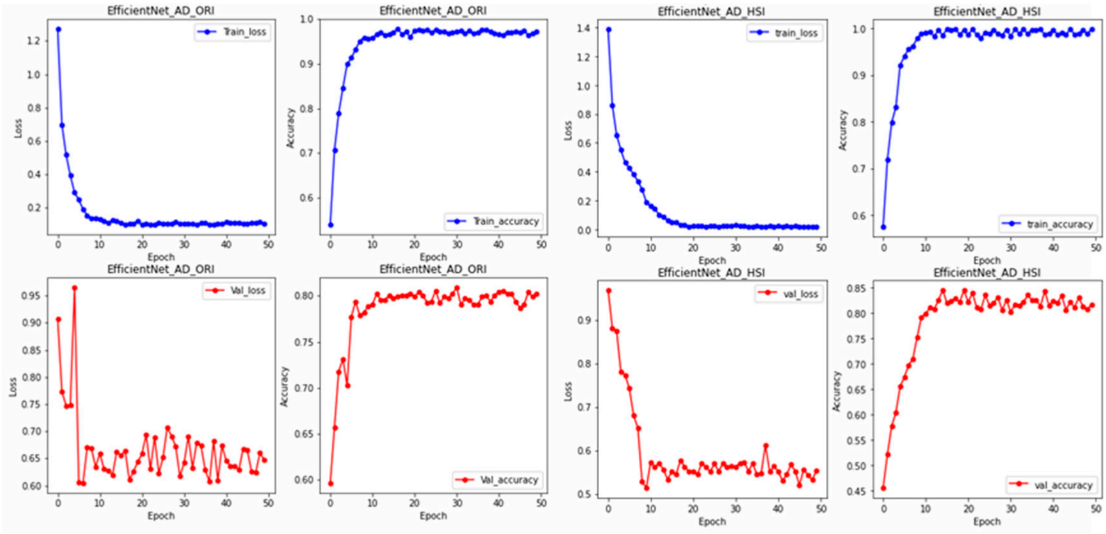


**Figure S14**. Loss and accuracy of EfficientNet_B0

## Reference

[40] Littmann, H. Die Zeiss-Funduskamera. *Ber* **1955**, *59*, 318.
